# Cell Populations Expressing Stemness-Associated Markers in Lung Adenocarcinoma

**DOI:** 10.3390/life11101106

**Published:** 2021-10-18

**Authors:** Claudia Paterson, Ethan J. Kilmister, Helen D. Brasch, Nicholas Bockett, Josie Patel, Erin Paterson, Gordon Purdie, Sean Galvin, Paul F. Davis, Tinte Itinteang, Swee T. Tan

**Affiliations:** 1 Gillies McIndoe Research Institute, Wellington 6242, New Zealand; claudiapatersonnz@gmail.com (C.P.); ethankilmister467@gmail.com (E.J.K.); helen.brasch@wellingtonscl.co.nz (H.D.B.); nick.bockett@gmri.org.nz (N.B.); josie.patel@gmri.org.nz (J.P.); erin.paterson@gmri.org.nz (E.P.); sean.galvin@ccdhb.org.nz (S.G.); paul.davis@gmri.org.nz (P.F.D.); tinte01@yahoo.com (T.I.); 2 Biostatistical Group, Dean’s Department, University of Otago, Wellington 6021, New Zealand; gordon.purdie@otago.ac.nz; 3 Department of Cardiothoracic Surgery, Wellington Regional Hospital, Wellington 6021, New Zealand; 4 Wellington Regional Plastic, Maxillofacial & Burns Unit, Hutt Hospital, Lower Hutt 5010, New Zealand; 5 Department of Surgery, The Royal Melbourne Hospital, The University of Melbourne, Melbourne, VIC 3010, Australia

**Keywords:** lung adenocarcinoma, stemness-associated markers, induced pluripotent stem cells, embryonic stem cells, cancer stem cells, treatment resistance, tumorsphere

## Abstract

The stemness-associated markers OCT4, NANOG, SOX2, KLF4 and c-MYC are expressed in numerous cancer types suggesting the presence of cancer stem cells (CSCs). Immunohistochemical (IHC) staining performed on 12 lung adenocarcinoma (LA) tissue samples showed protein expression of OCT4, NANOG, SOX2, KLF4 and c-MYC, and the CSC marker CD44. In situ hybridization (ISH) performed on six of the LA tissue samples showed mRNA expression of OCT4, NANOG, SOX2, KLF4 and c-MYC. Immunofluorescence staining performed on three of the tissue samples showed co-expression of OCT4 and c-MYC with NANOG, SOX2 and KLF4 by tumor gland cells, and expression of OCT4 and c-MYC exclusively by cells within the stroma. RT-qPCR performed on five LA-derived primary cell lines showed mRNA expression of all the markers except SOX2. Western blotting performed on four LA-derived primary cell lines demonstrated protein expression of all the markers except SOX2 and NANOG. Initial tumorsphere assays performed on four LA-derived primary cell lines demonstrated 0–80% of tumorspheres surpassing the 50 µm threshold. The expression of the stemness-associated markers OCT4, SOX2, NANOG, KFL4 and c-MYC by LA at the mRNA and protein level, and the unique expression patterns suggest a putative presence of CSC subpopulations within LA, which may be a novel therapeutic target for this cancer. Further functional studies are required to investigate the possession of stemness traits.

## 1. Introduction

Lung cancer affects 2.1 million people annually and was the cause of 1.7 million deaths worldwide in 2018 [1]. Lung cancer is classified as either small cell lung carcinoma or non-small cell lung carcinoma (NSCLC), which account for 15% and 85% of lung cancers, respectively [2]. NSCLC can be further sub-classified as large cell carcinoma, squamous cell carcinoma or adenocarcinoma [3].

Lung adenocarcinoma (LA) is the most common type of lung cancer, accounting for 40% of all lung cancers [2]. Although the overall incidence of lung cancer has been decreasing [4], the incidence of LA has been increasing since the 1950s [5]. LA affects females more than males and causes at least 80% of lung cancers [2]. LA is the most common lung cancer diagnosed in non-smokers [3].

LA is a small and slow growing gland-forming tumor often located in the periphery of the lung [6]. They are heterogenous and are subtyped in clinical settings depending on the predominant histological pattern observed which include lepidic, acinar, papillary, micropapillary and solid predominant categories [3].

Current treatment for NSCLC includes surgery for stage I, II or IIIa tumors that are deemed resectable [2]. Radiotherapy is used as a primary therapy for patients who are not surgical candidates, or for palliation [2]. Chemotherapy may be used as an adjunct to surgery, and is the first-line treatment for advanced cases, with platinum-based agents being the mainstay therapy [2]. Targeted therapy is used for individuals whose cancer contains certain gene mutations, such as *EGFR* and *K-Ras* mutations [6]. Despite treatment, the overall 5-year survival for NSCLC is 18% [2].

Numerous studies have reported the presence of stem-like subpopulations in NSCLC, using common cancer stem cell (CSC) markers such as CD44 [7,8,9,10], CD133 [11,12,13,14] and ALDH1 [15]. The association of the aforementioned CSC markers with cancer underscores better understanding of cancer biology and potential therapeutic target options.

Yamanaka et al. demonstrate that mouse [16] and human [17] adult fibroblasts can be reprogrammed to an embryonic stem cell (ESC)-like state using the four transcription factors OCT4, SOX2, KLF4 and c-MYC. These transcription factors are known to play an important role in pluripotency and immortality of stem cells during embryogenesis, and have been termed induced pluripotent stem cells (iPSCs). Interestingly, CSCs have been noted to have similar characteristics to ESCs [18]. Thomson et al. demonstrate that human somatic cells can be reprogrammed to form pluripotent stem cells using OCT4, SOX2, NANOG and another transcription factor called LIN28 [19]. In this study, we investigated the presence of transcription factors OCT4, SOX2, NANOG, KLF4 and c-MYC in LA and refer to these transcription factors as stemness-associated markers throughout this study.

OCT4 is a transcription factor fundamental for somatic cell reprogramming and stem cell pluripotency, and its overexpression has been associated with tumorigenicity and metastasis [20]. SOX2 is a transcription factor critical for maintaining stem cell pluripotency, and forms a complex with OCT4 which can act on downstream genes [21]. KLF4 is a transcription factor which appears to function as an oncogene and a tumor suppressor gene in different cancer types [22]. c-MYC is an oncogene involved in cell growth and differentiation, known to be upregulated in many cancer types [23]. NANOG is a transcription factor that interacts with OCT4 and SOX2, and is involved in induction of pluripotency [24]. However, it is not one of the four essential transcription factors required for generation of iPSCs [16].

Previous studies have shown OCT4 upregulation in CD133+ lung cancer cells [10] are associated with poor prognosis [25] and contribute to tumorigenesis and metastasis [26,27]. They also contribute to chemotherapy and radiotherapy resistance [10]. Research has shown overexpression of NANOG confers a worse prognosis [26,28], particularly when nuclear staining is observed [29,30]. For this reason, NANOG has been proposed as a prognostic marker [31,32]. NANOG is also thought to assist OCT4 in cancer and epithelial-to-mesenchymal transition in LA [28]. SOX2 upregulation has been demonstrated in LA and plays a role in maintaining stemness and tumorigenicity [33,34,35], and its expression is an indicator of poor prognosis [36]. Furthermore, overexpression of SOX2 alongside OCT4 has been shown to contribute to radiotherapy-resistance [37]. Some studies have found that KLF4 is downregulated in lung cancer, concluding a role in tumor suppression in lung tissue [38,39]. The function of KLF4 may depend on its subcellular location, with one study showing nuclear expression conferring a poor prognosis and cytoplasmic expression conferring a good prognosis for NSCLC [40]. c-MYC has been reported to be overexpressed in 20% of NSCLC and contributes to tumor progression [41]. However, further research is needed to understand the significance of the presence of stemness-associated markers on cells in LA to shed light on its pathogenesis.

The aim of this study was to demonstrate the expression of the stemness-associated markers OCT4, NANOG, SOX2, KLF4, c-MYC in LA. Numerous studies have investigated the expression of these markers singly. The aim of our study is to investigate expression of all of these markers simultaneously within the same LA tissue samples. Evidence shows these markers interact with one another in feedback loops within a network, and hence must be taken into account as a group of markers. To strengthen our study, we have utilized a variety of descriptive laboratory techniques to demonstrate the presence of these markers at both the transcriptional and translational levels. Given the common use of CD44 as a marker of CSCs in lung cancer research, we use immunohistochemical (IHC) staining of LA tissue samples, alongside the stemness-associated markers, in this study.

## 2. Materials and Methods

### 2.1. Lung Adenocarcinoma Tissue Samples

LA tissue samples from six male and six female patients, aged 49–77 (mean, 65.8) years (Table S1) were sourced from the Gillies McIndoe Research Institute Tissue Bank for this study. These LA tissue samples were donated by patients who underwent surgical resection of in Wellington, New Zealand. This study was approved by the Central Regional Health and Disability Ethics Committee (Ref. 18/CEN/184) with written consent from all participants.

### 2.2. Lung Adenocarcinoma-Derived Primary Cell Lines

Primary cell lines were derived from five available LA tissue samples from the original cohort of 12 patients, by culturing pieces of LA tissue between Matrigel layers (cat# 354234, Corning Life Sciences, Tewksbury, MA, USA) in 24-well plates (Raylab, Auckland, New Zealand) and adding an explant culture media consisting of DMEM (1X) and GlutaMAX-1 (cat# 10569010, Gibco, ThermoFisher Scientific, Waltham, MA, USA), supplemented with 2% penicillin/streptomycin (cat# 15140122, Gibco, ThermoFisher Scientific, Waltham, MA, USA) and 0.2% gentamicin/amphotericin B (cat# R01510, Gibco, ThermoFisher Scientific, Waltham, MA, USA). Cells were extracted from the Matrigel with Dispase (cat #354235, Corning Life Sciences, Tewksbury, MA, USA) following growth and transferred to a monolayer culture. The media for the monolayer culture consisted of DMEM (cat# 10569010, Gibco), 10% fetal calf serum (cat# 10091148, Gibco, ThermoFisher Scientific, Waltham, MA, USA), 5% mTeSR (cat# 85850, StemCell Technologies, Vancouver, BC, Canada), 1% penicillin/streptomycin (cat# 15140122, Gibco, ThermoFisher Scientific, Waltham, MA, USA) and 0.2% gentamicin/amphotericin B (cat# R01510, Gibco, ThermoFisher Scientific, Waltham, MA, USA). Cultures were maintained in a humidified incubator at 37 °C with 5% CO_2_ atmosphere, and all LA-derived primary cell lines used in experiments were between passages 4 and 7 with the exception of the initial tumorsphere formation assays which were between passage 8 and 11.

### 2.3. Histochemical and Immunohistochemical Staining

Hematoxylin and eosin (H&E) staining was performed on 4 µm-thick formalin-fixed paraffin-embedded sections of all 12 LA tissue samples, to confirm the diagnosis of LA by an anatomical pathologist. These sections underwent IHC staining as previously described [42], using the Leica BOND RX™ auto-stainer (Leica, Nussloch, Germany) with primary antibodies for stemness-associated markers OCT4 (1:30; cat# MRQ-10, Cell Marque, Rocklin, CA, USA), NANOG (1:200; cat# EP225, Cell Marque, Rocklin, CA, USA), SOX2 (1:500; cat# PA1-094, ThermoFisher Scientific, Rockford, IL, USA), KLF4 (1:100; cat# nBP2-24749, Novus Biologicus LLC, Littleton, CO, USA) and c-MYC (1:1000; cat# ab32, Abcam, Cambridge, MA, USA), and the CSC marker CD44 (1:1,500; cat# MRQ-13, Cell Marque, Rocklin, CA, USA), and 3,3′-diaminobenzidine as the chromogen. Antibodies were diluted with BOND™ primary antibody diluent (cat# AR9352, Leica, Nussloch, Germany), and slides were mounted in Surgipath Micromount (cat#38017322, Leica, Nussloch, Germany).

Three representative samples of LA from the original cohort of 12 patients underwent immunofluorescence (IF) staining, as previously described [42]. The same primary antibodies and concentrations were used in IHC staining. Dual-staining of OCT4 and c-MYC with NANOG, SOX2 and KLF4 was performed, with primary antibodies detected using a fluorescent secondary antibody of either Vectafluor Excel anti-mouse 488 (ready-to-use; cat# VEDK2488, Vector Laboratories, Burlingame, CA, USA) and Alex Fluor anti-rabbit 594 (1:500; cat# A21207, Life Technologies, Carlsbad, CA, USA). Slides were mounted in Vecta Shield Hardset mounting medium and counter-stained with 4′,6-diamino-2-phenylindone (cat# H-1500, Vector Laboratories, Burlingame, CA, USA).

Positive human control tissues used were tonsil for CD44, seminoma for OCT4 and NANOG, skin epidermis for SOX2, breast carcinoma for KLF4 and colon mucosa for c-MYC. Negative controls were performed on sections of LA using a matched isotype control for mouse (ready-to-use; cat# IR750, Dako, Carpinteria, CA, USA) and rabbit (ready-to-use; cat# IR600, Dako, Carpinteria, CA, USA) primary antibodies, and a combination for IF staining.

### 2.4. Image Capture and Analysis

IHC-stained slides were viewed and imaged using an Olympus BX53 microscope and an Olympus SC100 digital camera and the cellSens 2.0 software (Olympus, Tokyo, Japan). IF-stained slides were viewed and imaged using an Olympus FV1200 biological confocal laser-scanning microscope, and images were processed with cellSens Dimension 1.11 software using 2D deconvolution algorithm (Olympus, Tokyo, Japan).

### 2.5. In Situ Hybridization

4 µm-thick formalin-fixed paraffin-embedded sections of six LA tissue samples from the original cohort of 12 patients underwent in situ hybridization (ISH) staining, on the Leica BOND™ RX auto-stainer with probes for c-MYC (NM_002467.4), KLF4 (NM_001314052), NANOG (NM_024865.2), OCT4 (NM_002701.4) and SOX2 (NR_075091.1), as previously described [42]. All probes were obtained from Advanced Cell Diagnostics (Newark, CA, USA) and were detected using the RNAscope 2.5 LS Reagent Brown Kit (cat# 322100, Advanced Cell Diagnostics, Newark, CA, USA).

Positive human controls included seminoma for OCT4 and NANOG, skin epidermis for SOX2, breast carcinoma for KLF4 and prostatic tissue for c-MYC. Negative controls were demonstrated on LA sections using a probe for DapB (EF191515; cat# 3120358, Advanced Cell Diagnostics, Newark, CA, USA).

### 2.6. Cell Counting and Statistical Analysis

Counting of cells in the tumor glands or those in the stroma with positive nuclear or cytoplasmic staining for CD44, OCT4, NANOG, SOX2, KLF4 and c-MYC was performed in six representative fields of view at 400x magnification. This was performed on each of the 12 LA tissue samples that underwent IHC staining, using Cell Counter on ImageJ software (LOCI, University of Wisconsin, WI, USA). A cell stained positive for OCT4, SOX2, NANOG, KLF4 and c-MYC if it resembled the positive control for that marker and was deemed negative for staining if it did not. The subcellular localization of the staining was also noted, given the staining indicating a positive cell could be located in either the nucleus or cytoplasm. Cells were distinguished from one another by the presence of their nuclei and counted. Cell counting for ISH-stained slides was performed at 1,000x magnification for OCT4, SOX2, NANOG, KLF4 and c-MYC in six representative fields of view for each of the six samples that underwent ISH staining.

For each marker, positive-stained cells were counted and the proportions of positive-stained cells out of the total number of cells within the field of view were then calculated and averaged across the six fields of view that had a minimum of 100 cells per field.

The proportion of cells with protein expression demonstrated by IHC staining and mRNA expression by ISH was analyzed with a mixed model logistic regression. A fixed term for marker and random terms for patient, patient x marker and slide within patient were used. Adjustment was made for multiple comparisons with the Holm–Bonferroni method. The glimmix procedure in the SAS 9.4 package (SAS Institute Inc., Cary, NC, USA) was used. Where no evidence of protein expression or mRNA expression was found for a marker, the patient’s data was excluded for that marker.

### 2.7. RT-qPCR and Statistical Analysis

Total RNA was extracted from frozen pellets of 5 × 10^5^ viable cells per sample from five LA-derived primary cell lines from the original cohort of 12 patients using the RNeasy Micro Kit (cat# 74004, Qiagen, Germantown, MD, USA). A DNase digest step was included to remove DNA contamination (cat# 79254, Qiagen, Germantown, MD, USA). RNA quantity was determined using a NanoDrop 2000 Spectrophotometer (Thermo Fisher Scientific, Waltham, MA, USA). Transcriptional expression was analyzed using the Rotor-Gene Q (Qiagen, Germantown, MD, USA) and the Rotor-Gene Multiplex RT-PCR Kit (cat# 204974, Qiagen, Germantown, MD, USA), using 40ng RNA samples, in triplicates. The TaqMan Gene Expression Assay primer probes (cat# 4331182, Thermo Fisher Scientific, Waltham, MA, USA) used included OCT4 (Hs03005111_g1), SOX2 (Hs00602736_s1), NANOG (Hs02387400_g1), KLF4 (Hs00358836_m1), c-MYC (Hs00153408_m1), and the reference genes GAPDH (Hs99999905_m1) and PUM1 (Hs00206469_m1). Universal human reference RNA (UHR; cat# CLT636690, Takara, Shiga, Japan), total RNA from healthy donor tissues, was used as the calibrator for the ΔΔCt analysis following normalization to the reference genes. Graphs were generated using GraphPad Prism (v8.0.2, San Diego, CA, USA) and results presented as fold-change, relative to UHR.

NTERA-2 cells were used as a positive control, no template controls (NTC) were performed to assess for contamination, and no reverse transcriptase controls (No RT) included for those assays which may detect genomic DNA. Probe specificity was confirmed by running end-point amplification products on 2% agarose gels (cat# G402002, Thermo Fisher Scientific, Waltham, MA, USA).

ΔΔCt was analyzed with a mixed model analysis of variance with a term for marker and a random term for cell line with a different variance for each marker. Adjustment was made for multiple comparisons with the Holm–Bonferroni method. The mixed procedure in the SAS 9.4 package (SAS Institute Inc., Cary, NC, USA) was used.

### 2.8. Western Blotting

Total protein was extracted from four LA-derived primary cell lines. Protein was separated by SDS-PAGE and transferred to a PVDF membrane as previously described [43]. The iBind Flex (cat# SLF2000, ThermoFisher Scientific, Waltham, MA, USA) was used for antibody binding with the primary antibodies for OCT4 (1:1000; cat# ab109183, Abcam, Cambridge, UK), NANOG (1:1000; cat# ab109250, Abcam, Cambridge, UK), SOX2 (1:500; cat# 48-1400, ThermoFisher Scientific, Waltham, MA, USA), KLF4 (1:000; cat# NBP2-24749, Novus Biologicus, Centennial, CO, USA), c-MYC (1:1000; cat# ab32072, Abcam, Cambridge, UK) and α-tubulin (1:2000; cat# 62204, ThermoFisher Scientific, Waltham, MA, USA). Secondary antibodies used included goat anti-rabbit HRP (1:1000; cat# ab6721, ThermoFisher Scientific, Waltham, MA, USA) and goat anti-mouse alexa488 (1:1000; cat# A21202, ThermoFisher Scientific, Waltham, MA, USA). Clarity Western ECL (cat# 1705061, Bio-Rad, Los Altos, CA, USA) was used for visualizing HRP detected protein bands and the ChemiDoc MP Imaging System (Bio-Rad Laboratories, Los Altos, CA, USA) and Image Lab 6.0 software (Bio-Rad Laboratories, Los Altos, CA, USA) were used for band detection and analysis. NTERA-2 cells were used as a positive control, and α-tubulin was used as a loading control.

### 2.9. In Vitro Initial Tumorsphere Formation Assays

Four adherent LA-derived primary cell lines were seeded for tumorsphere formation. StemXVivo serum-free tumorsphere media (cat# CCM012, R&D Systems, Minneapolis, MN, USA) was supplemented with 2U/mL Heparin (cat# 2812, Tocris, Auckland, New Zealand) and 0.5 µg/mL Hydrocortisone (cat# 4093, Tocris Bioscience, Bristol, UK). T75 Nunclon™ Sphera™ EasYFlasks (cat# 174952, ThermoFisher Scientific, Waltham, MA, USA) were seeded with 24 mL of media containing 1 × 10^4^ live cells per mL as determined by cell counting using the Trypan Blue Exclusion method. They were incubated at 37 °C with 5% CO_2_ for 10 days or until initial signs of dark necrotic centers were observed. Feeding occurred every three to four days by the addition of 12 mL of tumorsphere media. Daily measuring was performed from day three by taking three representative images for each cell line. Five typical sphere-like structures from each image were measured in the longest and shortest dimension, and measurements used to establish a nominal average size for each sphere.

A cell line was considered positive for initial tumorsphere formation if the average sphere size was greater than 50 µm, and if at least 50% of measured spheres were greater than 50 µm, according to previously established criteria [44].

## 3. Results

### 3.1. IHC Staining Demonstrated the Presence the Cancer Stem Cell Marker CD44, and the Five Stemness-Associated Markers in Lung Adenocarcinoma Tissues

**Figure 1:**
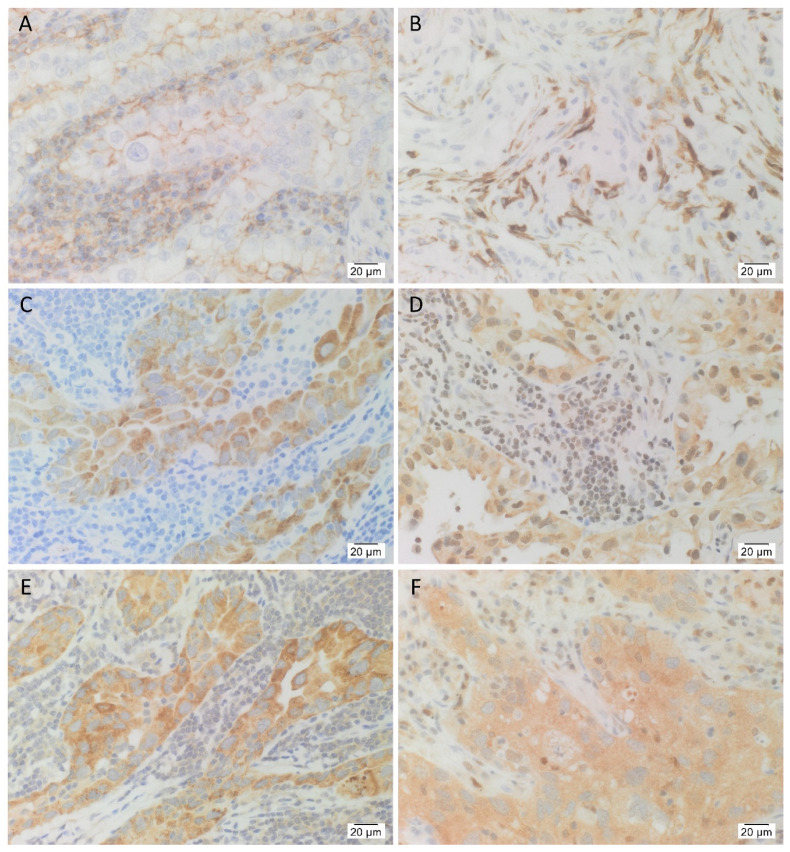
Representative immunohistochemical-stained images of lung adenocarcinoma (LA) tissue samples demonstrating the expression of CD44, OCT4, NANOG, SOX2, KLF4 and c-MYC. LA was organized into tumor glands surrounded by the adjacent stroma. Membranous staining of CD44 ((**A**), brown) was consistently observed on the cells within the tumor glands, and some cells within the stroma. OCT4 ((**B**), brown) was consistently expressed in the cytoplasm of cells within the stroma, and occasionally, in the nucleus of cells within the stroma and the cytoplasm of cells within the tumor glands. NANOG ((**C**), brown) was consistently seen in the cytoplasm of cells within the tumor glands. SOX2 ((**D**), brown) was observed in the nucleus and the cytoplasm of cells within the tumor glands, as well as the nucleus of cells within the stroma. KLF4 ((**E**), brown) was consistently seen in the cytoplasm of cells within the tumor glands, and also in the cytoplasm of cells within the stroma. c-MYC ((**F**), brown) was observed in the nucleus and the cytoplasm of cells within the tumor glands, and the cells within the stroma. Nuclei were counterstained with hematoxylin ((**A**–**F**), blue). Original magnification: 400×; *n* = 12.

H&E staining confirmed the presence of LA in all 12 samples (Figure S1). IHC-stained sections of the same 12 LA tissue samples demonstrated consistent membranous staining of the CSC marker CD44 in tumor cells, as well as membranous and cytoplasmic staining in stromal cells (Figure 1A). All 12 cases stained positively for OCT4 (Figure 1B). It was consistently seen in the cytoplasm of stromal cells adjacent to tumor glands, and in the nucleus of stromal cells in two of 12 cases, as well as the cytoplasm of tumor cells in three of the 12 cases. Eight of the 12 cases stained positively for NANOG (Figure 1C) and this was consistently seen in the cytoplasm of tumor cells. Ten of the 12 cases stained positively for SOX2 (Figure 1D) which was seen in the nucleus and the cytoplasm of tumor cells, as well as the nucleus of stromal cells adjacent to tumor glands, in nine of 10 cases. All 12 cases stained positively for KLF4 (Figure 1E) which was consistently seen in the cytoplasm of tumor cells and the cytoplasm of stromal cells adjacent to tumor glands in six of the 12 cases. All 12 cases stained positively for c-MYC (Figure 1F) in the nucleus of tumor cells in 10 of 12 cases, the cytoplasm of tumor cells in nine of the 12 cases, the nucleus of stromal cells in nine of the 12 cases, and the cytoplasm of stromal cells in six of the 12 cases.

**Figure 2:**
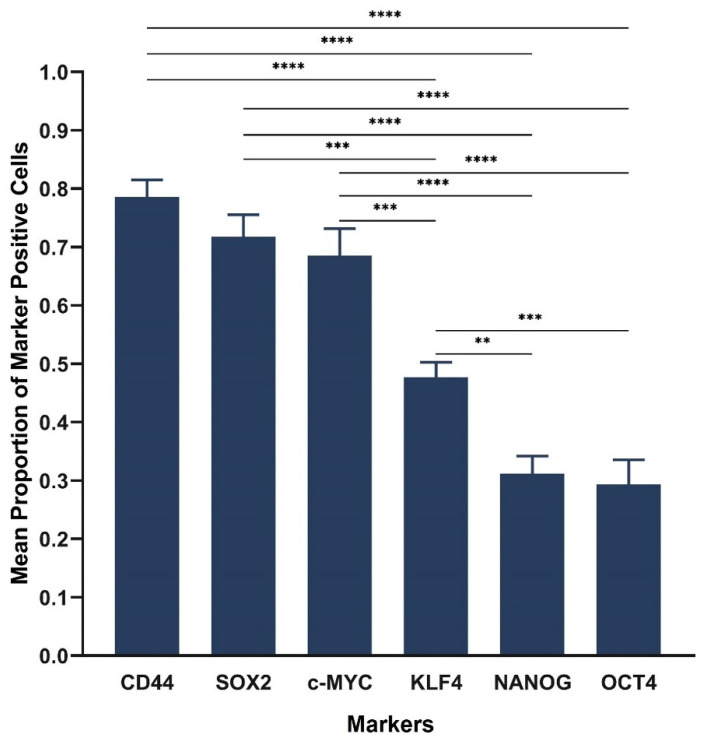
Statistical analyses of cell counting in 12 lung adenocarcinoma tissue samples. The graph demonstrates statistically significant differences between the mean proportion of cells stained positively for the cancer stem cell marker CD44 and each of the stemness-associated markers. All markers were significantly different except SOX2, c-MYC and CD44 which were not significantly different, nor were OCT4 and NANOG. CD44 had the highest mean proportion of positive cells, followed by SOX2, c-MYC, KLF4, NANOG and OCT4. Error bars: 95% confidence interval. ** *p* < 0.01; *** *p* < 0.001; **** *p* < 0.0001.

Cell counting of IHC-stained slides demonstrated the average proportion of cells expressing the CSC marker CD44 as 0.78, and the stemness-associated markers as 0.72 for SOX2, 0.67 for c-MYC, 0.47 for KLF4, 0.31 for NANOG and 0.29 for OCT4 (Figure 2). Statistical analysis of IHC cell counting results showed significant differences between the markers (F = 30.6, d.f. = 5,49, *p* < 0.0001). All markers showed significant differences except SOX2, c-MYC and CD44 which were not significantly different, nor were OCT4 and NANOG. The proportion of samples with evidence of protein expression was as follows: 100% (95% CI, 74–100%) for OCT4, KLF4, c-MYC and CD44; 83% (95% CI, 52–98%) for SOX2 and 67% (95% CI, 35–90%) for NANOG.

Positive staining was demonstrated on sections of control human tissues: tonsil for CD44 (Figure S2A), seminoma for OCT4 (Figure S2B) and NANOG (Figure S2C), skin epidermis for SOX2 (Figure S2D), breast carcinoma for KLF4 (Figure S2E), and colon mucosa for c-MYC (Figure S2F). Specificity of staining was confirmed on sections of LA tissues using a matched isotype control for both mouse and rabbit primary antibodies (Figure S2G).

### 3.2. Immunofluorescence Staining Showed Co-Expression of OCT4 and c-MYC with NANOG, SOX2 and KLF4 by Tumor Gland Cells, and Exclusive Expression of OCT4 and c-MYC by Stromal Cells in Lung Adenocarcinoma Tissues

**Figure 3:**
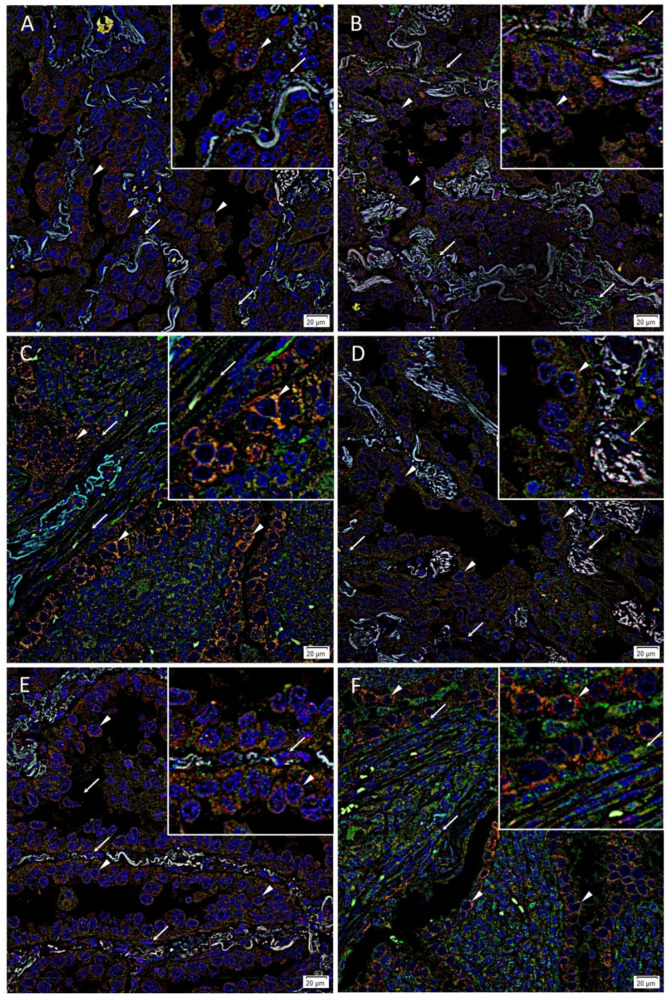
Representative immunofluorescence-stained images of lung adenocarcinoma tissue samples demonstrating expression of stemness-associated markers. OCT4 ((**A**–**C**), green) and NANOG ((**A**), red), SOX2 ((**B**), red) and KLF4 ((**C**), red) were expressed on the cells within the tumor glands (arrowheads). The expression pattern was predominantly cytoplasmic and was observed within the nucleus of some cells. OCT4 ((**C**), green) was exclusively expressed by cells within the stroma adjacent to tumor glands (arrows). c-MYC ((**D**–**F**), green) and NANOG ((**D**), red), SOX2 ((**E**), red) and KLF4 ((**F**), red) was expressed by cells within the tumor glands (arrowheads). The expression pattern was predominantly cytoplasmic and was observed within the nucleus of some cells. c-MYC ((**F**), green) was exclusively expressed by cells within the stroma adjacent to tumor glands (arrows). Nuclei were counterstained with 4′,6′-diamidino-2-phenylindole ((**A**–**F**), blue). Original magnification: 400×; *n* = 3. The insets show enlarged views of the corresponding images.

IF staining demonstrated co-expression of OCT4 (Figure 3A–C, green) with NANOG (Figure 3A, red), SOX2 (Figure 3B, red) and KLF4 (Figure 3C, red) by cells within the tumor glands (arrowheads). OCT4 (Figure 3A–C, green) was also expressed by some cells within the stroma (arrows). c-MYC (Figure 3D–F, green) was also co-expressed with NANOG (Figure 3D, red), SOX2 (Figure 3E, red) and KLF4 (Figure 3F, red) by cells within the tumor glands (arrowheads). c-MYC (Figure 3D–F, green, arrows) was also expressed by some cells within the stroma. Magnified figure insets have been provided to show enlarged views of the corresponding images. Split images of IF staining presented in Figure 3 are shown in Figure S3. The negative controls demonstrated minimal staining (Figure S3M).

### 3.3. In Situ Hybridization Showed mRNA Expression of All Five Stemness-Associated Markers in Lung Adenocarcinoma Tissues

**Figure 4:**
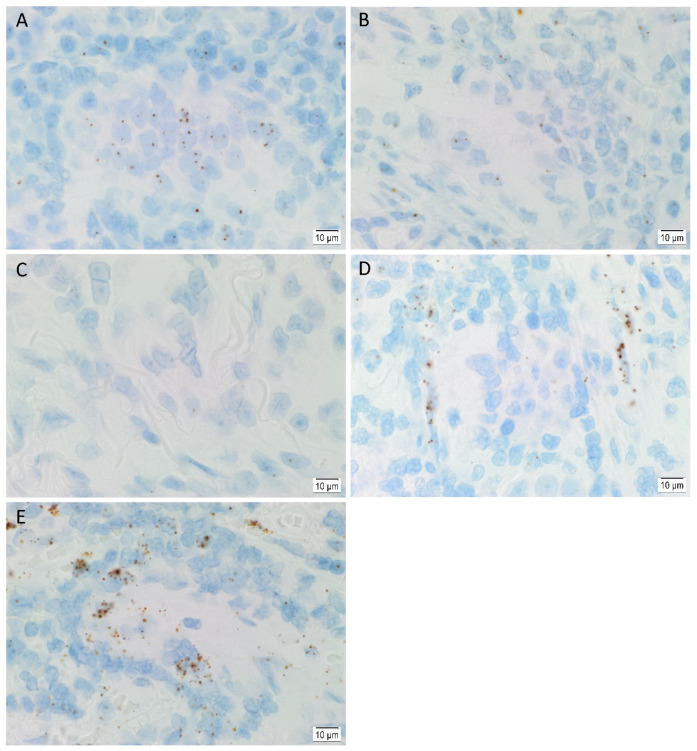
Representative in situ hybridization images of lung adenocarcinoma tissue samples demonstrating expression of mRNA transcripts for stemness-associated markers. Expression of OCT4 ((**A**), brown), NANOG ((**B**), brown), SOX2 ((**C**), brown), KLF4 ((**D**), brown) and c-MYC ((**E**), brown) can be observed at the mRNA level. Nuclei were counterstained with hematoxylin ((**A**–**E**), blue). Original magnification: 1000×; *n* = 6.

ISH demonstrated mRNA expression of OCT4 (Figure 4A), NANOG (Figure 4B), SOX2 (Figure 4C), KLF4 (Figure 4D) and c-MYC (Figure 4E) in all six LA tissue samples.

**Figure 5:**
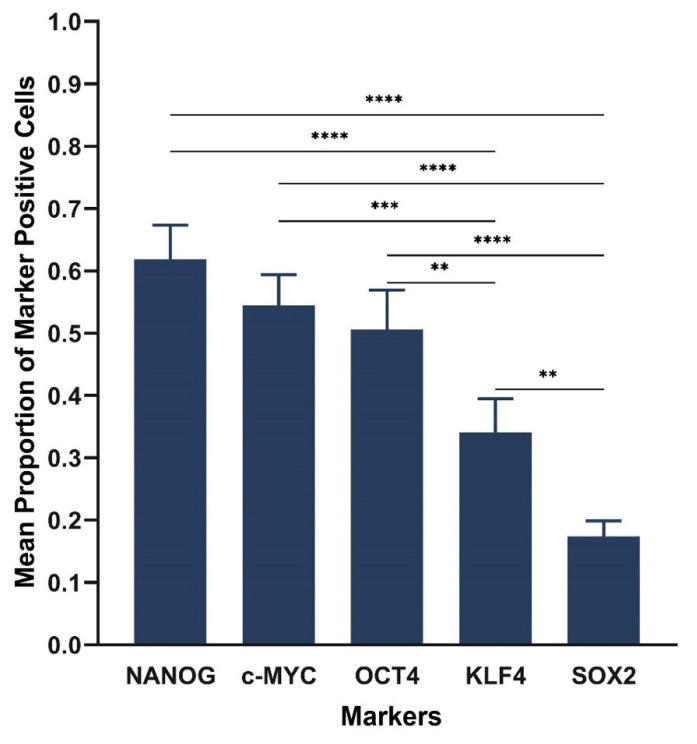
Statistical analyses of cell counting results for in situ hybridization stained slides of six lung adenocarcinoma tissue samples. The graph demonstrates statistically significant differences between the mean proportion of cells stained positively for each stemness-associated marker (*p <* 0.0001). There were significant differences between all the stemness-associated markers except OCT4, NANOG and c-MYC which were not significantly different. NANOG had the highest mean proportion of positive cells, followed by c-MYC, OCT4, KLF4 and SOX2. Error bars: 95% confidence interval. ** *p* < 0.01; *** *p* < 0.001; **** *p* < 0.0001.

Cell counting of ISH-stained slides demonstrated the average proportion of cells expressing markers as 0.62 for NANOG, 0.55 for c-MYC, 0.51 for OCT4, 0.34 for KLF4 and 0.17 for SOX2 (Figure 5). Statistical analysis of ISH cell counting results showed significant differences between the markers (F = 23.6, d.f. = 4,20, *p* < 0.0001). There were significant differences between all markers except OCT4, NANOG and c-MYC which did not reach a statistically significant difference. The proportion of patients with evidence of mRNA expression was 100% (95% CI, 54–100%) for each marker.

Expected staining patterns were demonstrated in control human tissues: seminoma for OCT4 (Figure S4A), normal skin for SOX2 (Figure S4B), seminoma for NANOG (Figure S4C), breast cancer for KLF4 (Figure S4D) and normal prostatic tissue for c-MYC (Figure S4E). Negative controls displayed minimal staining (Figure S4F).

### 3.4. RT-qPCR Demonstrated mRNA Expression of All Stemness-Associated Markers, except for SOX2 in Lung Adenocarcinoma-Derived Primary Cell Lines

**Figure 6:**
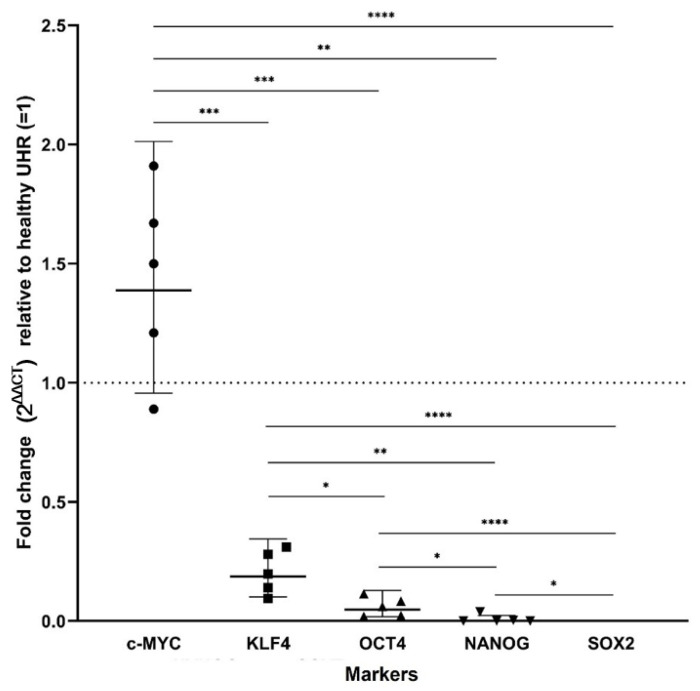
RT-qPCR data demonstrating the presence of mRNA transcripts of stemness-associated markers. The graph demonstrates 2^ΔΔCT^ values of RT-qPCR runs performed on five primary lung adenocarcinoma-derived primary cell lines demonstrating the presence of mRNA transcripts for c-MYC, KLF4, OCT4 and NANOG. SOX2 mRNA transcripts were not detected. ΔΔCT was calculated by normalizing CT values of stemness-associated markers to that of housekeeping genes GAPDH and PUM1, and then expressing this relative to the ΔCT of normal universal human reference RNA (UHR). * *p* <0.05; ** *p* < 0.01; *** *p* < 0.001; **** *p* < 0.0001.

Using primary cell lines derived from five of the 12 LA tissue samples, RT-qPCR demonstrated mRNA expression of c-MYC, KLF4, OCT4 and NANOG relative to healthy UHR, with SOX2 below detectable levels (Figure 6). c-MYC was increased above the baseline of 1 of UHR, but not above a 2-fold increase. KLF4, OCT4 and NANOG were below 0.5 of baseline, hence a greater than 2-fold decrease, meaning less mRNA was detected for these markers in the LAPCLs compared with the healthy UHR. The expected size amplicons were observed, and no products were observed in the no template control (NTC) and no reverse transcriptase control (No RT) reactions (Figure S5). This data suggests a hierarchy from most to least abundant of c-MYC, KLF4, OCT4, NANOG and SOX2. Statistical analyses demonstrated that all differences between the markers were statistically significant after adjustment was made for multiple comparisons with the Holm–Bonferroni method (F = 934.3, d.f. = 4,6.9, *p* < 0.0001). For SOX2, no amplification cycle crossed the threshold, consequently a value of 43 was used as a minimum.

### 3.5. Western Blotting Demonstrated the Presence of All Five Stemness-Associated Markers, except for NANOG in Lung Adenocarcinoma-Derived Primary Cell Lines

**Figure 7:**
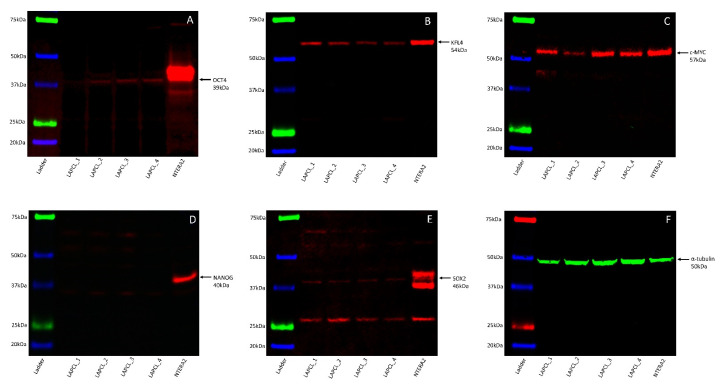
Representative Western blotting images of total protein extracted from four lung adenocarcinoma-derived primary cell lines demonstrating protein expression of stemness-associated markers. Arrows indicate the presence of the proteins with expected band sizes for OCT4 (**A**), KLF4 (**B**) and c-MYC (**C**). NANOG (**D**) and SOX2 (**E**) were below detectable levels. Bands for α-tubulin (**F**) confirmed approximately equal protein loading.

WB on the four LA-derived primary cell lines demonstrated expression of OCT4 in three out of four samples at approximately 39 kDa (Figure 7A), KLF4 in all four samples at approximately 54 kDa (Figure 7B), and c-MYC in all four samples at approximately 57 kDa (Figure 7C). SOX2 (Figure 7D) and NANOG (Figure 7E) were below detectable levels in all four samples but was detected in the NTERA-2 cells at the expected molecular weights of approximately 46 kDa (Figure 7D) and 40 kDa (Figure 7E), respectively. α-Tubulin confirmed approximate equivalent protein loading for all four LA-derived primary cell lines at approximately 50 kDa (Figure 7F). The NTERA-2 cells confirmed specificity for the five stemness-associated markers. The full-length WB images are shown in Figure 1 are presented in Figure S6.

### 3.6. In Vitro Initial Tumorsphere Formation Assays Demonstrated Evidence of Tumorspheres across Four Lung Adenocarcinoma-Derived Primary Cell Lines

**Figure 8:**
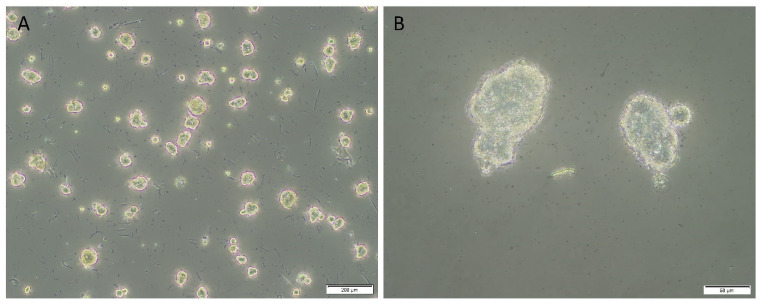
Representative low magnification (**A**) and high magnification (**B**) images of positive initial tumorsphere formation from the first lung adenocarcinoma-derived primary cell line (LAPCL). Initial tumorspheres derived from the first LAPCL averaged 63.81 µm (median, 66.19; range 43.52–85.28) in diameter at point of harvest after three days, and 80% of spheres had surpassed the 50 µm threshold. The second, third and fourth LAPCL had 0%, 17.6% and 13% of spheres surpass the 50 µm threshold, respectively. Original magnifications: 5× (**A**); 20× (**B**).

The first LA-derived primary cell line demonstrated evidence of positive initial tumorsphere formation. Eighty percent of the spheres passed the 50 µm threshold after three days (Figure 8A,B), with early signs of dark necrotic centers, and the spheres had an average diameter of 63.81 µm. Measurement of the second LA-derived primary cell line demonstrated that 0% of spheres measured had passed the 50 µm threshold after 10 days, and the spheres had an average diameter of 29.06 µm. Measurement of the third LA-derived primary cell line demonstrated that 17.6% of spheres measured had passed the 50 µm threshold after 10 days. The spheres had an average diameter of 49.71 µm. Measurement of the fourth LA-derived primary cell line demonstrated that 13% of spheres measured had passed the 50 µm threshold after 10 days with an average diameter of 38.90 µm. Details of the in vitro initial tumorsphere assays results are presented in Table 1.

## 4. Discussion

There is increasing evidence of cell populations expressing stemness-associated markers in LA. The CSC concept of cancer proposes that there is a small subpopulation of CSCs within the tumor, which divide asymmetrically to produce pluripotent CSCs and differentiated cancer cells which form the bulk of the tumor [45]. This hierarchical model of cancer accounts for the cellular heterogeneity observed within the tumor [46]. A hallmark of CSCs is their resistance to chemotherapy and radiotherapy [9] which result in the death of rapidly dividing differentiated cancer cells, but not CSCs, leading to subsequent tumor recurrence and/or metastases [33].

Previous research has shown that it is possible to induce somatic cells into an ESC-like state by introducing four critical transcription factors OCT4, SOX2, KLF4 and c-MYC [18]. These markers are expressed by ESCs during embryogenesis and imbue the cells with stemness, pluripotency and self-renewal [45]. We propose that the presence of these stemness-associated markers in LA tissues suggests a significant role in cancer development and progression. This is in line with a recent study investigating tumorsphere formation in NSCLC patients, and analyzed gene expression profiles of NANOG, NOTCH3, CD44, CDKN1A, SNAI1 and ITGA6. The authors formulate a gene expression score, termed ‘CSC score’, comprising a combination of some of the aforementioned genes, and they find that patients with high CSC scores have a shorter overall survival [47]. Our study demonstrates the presence of the aforementioned stemness-associated markers at the mRNA and protein levels within LA tissue samples, and primary cell lines derived directly from some of these tissues.

IHC staining confirmed protein expression of all five stemness-associated markers and the CSC marker CD44 [10], in nuclear and cytoplasmic locations within the tumor and stromal cells, depending on the marker and patient sample. Recent research has demonstrated how subcellular localization of these markers leads to downstream consequences on cellular functioning [24]. Compared with cytoplasmic expression, nuclear expression of some of the stemness-associated markers has been shown to be correlated with a worse prognosis [40]. This warrants further investigation.

IF staining showed co-expression of OCT4 and c-MYC with NANOG, SOX2 and KLF4 providing further evidence of the presence of the stemness-associated markers, as it would be expected that these cells would express multiple markers within a given cell. Furthermore, there was exclusive expression of OCT4 and c-MYC in the cytoplasm of stromal cells. The demonstration of cell subpopulations, expressing either multiple markers by the tumor cells or exclusively OCT4 and c-MYC by the stromal cells, suggests these subpopulations may be related to one another. It is exciting to speculate that this stromal subpopulation may play a role early in the development of LA. These subpopulations may represent various stages of differentiation, with cells exclusively expressing OCT4 and c-MYC sitting atop a cellular hierarchy, being less differentiated. This is consistent with our previous publications suggesting OCT4+ cells represent the most primitive cell population in glioblastoma [47,48].

ISH demonstrated mRNA expression for all five stemness-associated markers in all six LA tissue samples investigated. Interestingly, SOX2 mRNA expression was observed at low levels compared with the other stemness-associated markers. However, IHC staining demonstrated ubiquitous staining of SOX2 throughout the nucleus and cytoplasm of tumor cells, and in the nucleus of stromal cells. This discrepancy between SOX2 mRNA and protein expression is intriguing and warrants further investigation.

RT-qPCR confirmed mRNA expression of c-MYC, KLF4, OCT4 and NANOG in all five LA-derived primary cell lines. SOX2 was below detectable levels, which correlated with the ISH data demonstrating low semi-quantitative mRNA expression of SOX2.

WB confirmed the presence of protein for OCT4, KLF4 and c-MYC, with OCT4 shown at low levels in three of the four LA-derived primary cell lines. This suggests a relatively low protein expression of OCT4, consistent with OCT4 sitting atop a cellular hierarchy. It may also be caused by sampling bias given a small sample size of four LA-derived primary cell lines generated from our cohort of 12 LA tissue samples being available for WB analysis. SOX2 and NANOG being below detectable levels in all four LA-derived primary cell lines, while they were demonstrated in LA tissues at the protein level by IHC staining, may be due a proportion of cells that expressed these markers not surviving the culture process. It may also result from sampling bias, or that WB is not sufficiently sensitive to detect protein expression of SOX2 and NANOG within the four LA-derived primary cell lines.

The four LA-derived primary cell lines that underwent assessment for potential tumorsphere formation demonstrated a range from 0% to 80% of measured sphere-like structures that passed the minimum 50 µm measurement threshold for a “positive” potential tumorsphere [44]. The first LA-derived primary cell line satisfied both criteria of an average sphere size greater than 50 µm, and at least 50% of measured spheres were greater than 50 µm, while the remaining three LA-derived primary cell line failed to satisfy these criteria. A possible explanation for this result is that few or no cells capable of forming tumorspheres were seeded from the adherent cultures for these three LA-derived primary cell lines. A small number of cells with tumorsphere potential may account for the variation in the percentage of “positive” tumorspheres between the cell lines. This preliminary data requires further validation with larger samples size and additional passaging to conclusively determine the functional capacity of the LA-derived primary cell lines. Multiple sphere forming passages would be required to demonstrate true tumorsphere positivity in a given cell line.

Previous research has demonstrated an association between overexpression of stemness-associated markers and aggressiveness of cancer. The differences in the expression of the stemness-associated markers may be reflected across a spectrum of disease aggression in LA. However, the small sample size of LA tissue samples included in this study precludes a firm conclusion on the relative abundance of stemness-associated markers and the clinical outcomes. Further research using a larger sample size would enable analysis of the impact of different histological subtypes and the degree of differentiation. The expression of OCT4 on CD133+ cells in the lung cancer is associated with tumorigenesis and metastasis, and overexpression of NANOG is associated with a worse prognosis [14,26,28]. Given prior research has identified the significant interplay between stemness-associated markers, it would be interesting to further understand their interactions and how this relates to the pathogenesis of cancer. Nevertheless, across the available 12 LA tissue samples, we were able to demonstrate the presence of all five stemness-associated markers at the mRNA and protein levels.

In conclusion, this study demonstrates the expression of the five stemness-associated markers in LA tissue samples and some of these markers in primary cell lines derived directly from some of these tissue samples, at the mRNA and protein level. The detection of stemness-associated markers provides preliminary evidence of the presence of CSC subpopulations within LA, which needs to be further investigated with functional studies. These markers are expressed on the cells within the tumor glands, as well as those within the stroma, in varying patterns of expression depending on the marker and patient sample. We propose that the possible CSC subpopulations within the tumor glands and the other within the stroma are related to each other, and may give clues to the pathogenesis of LA. These cell subpopulations expressing stemness-associated markers within LA may be a novel therapeutic target for the treatment for this aggressive cancer. It has been proposed that CSCs expressing stemness-associated markers may be targeted using medications that inhibit the renin-angiotensin system and its converging pathways [49,50]. Future investigations into therapeutic targeting of these CSCs by drug-repurposing has the potential to improve morbidity and mortality of patients with LA.

## Figures and Tables

**Table 1:** In vitro tumorsphere formation assays on four lung adenocarcinoma-derived primary cell lines.

Cell Line	Initial Tumorsphere Size (µm) *			% of Initial Tumorspheres > 50 µm	Days for Initial Signs of Dark Centers
	Mean	Median	Range		
1	63.81	66.19	43.52–85.28	80	3
2	29.06	27.01	20.91–43.02	0	ns **
3	49.71	40.12	29.12–122.05	17.6	10
4	38.90	38.07	26.78–60.95	13	ns **

* Measurements taken on the final day in culture. ** No dark centers observed at the completion of the full 10-day tumorsphere culture period.

## Data Availability

Publicly available datasets were analyzed in this study. This data can be found here: Paterson, Claudia (2021): Cell Populations Expressing Stemness-Associated Markers in Lung Adenocarcinoma. figshare. Dataset. https://doi.org/10.6084/m9.figshare.16822918.v3.

## Supplementary Materials

The following are available online at https://www.mdpi.com/article/10.3390/life11101106/s1, Table S1 Patient characteristics and smoking status. Figure S1: Representative hematoxylin and eosin images of lung adenocarcinoma demonstrating the tumor glands surrounded by stroma. Nuclei were counterstained with hematoxylin ((A,B), blue). Original magnifications: 100× (A) and 400× (B). Figure S2: Representative positive control immunohistochemical-stained images demonstrating the expression of CD44 ((A), brown) in tonsil, OCT4, ((B), brown) and NANOG ((C), brown) in seminoma, SOX2 ((D), brown) in skin epidermis, KLF4 ((E), brown) in breast carcinoma, and c-MYC ((F), brown) in normal colon mucosa. A negative control (G). Nuclei were coun-terstained with hematoxylin (A–G, blue). Original magnification: 400×. Figure S3: Split immunofluorescence-stained images shown in Figure 3 demonstrating the expression of NANOG ((A), red) and OCT4 ((B), green); SOX2 ((C), red) and OCT4 ((D), green); KLF4 ((E), red) and OCT4 ((F), green); NANOG ((G), red) and c-MYC ((H), green); SOX2 ((I), red) and c-MYC ((J), green); KLF4 ((K), red) and c-MYC ((L), green). A negative control (M). Cell nuclei were counterstained with 4’,6 diamidino-2-phenylindole ((A–M), blue). Original magnification: 400×. Figure S4: Representative in situ hybridization positive controls for OCT4 ((A), brown) and NANOG ((B), brown) in seminoma, SOX2 ((C), brown) in normal skin, KLF4 ((D), brown) breast carcinoma and c-MYC ((E), brown) in normal prostatic tissue. Nega-tive controls (F) performed on sections of lung adenocarcinoma tissue samples confirmed specificity of secondary anti-body. Nuclei were counterstained with hematoxylin ((A–F), blue). Original magnification: 1000×. Figure S5: RT-qPCR amplification products from five lung adenocarcinoma-derived primary cell lines. The specificity of the primer probes was confirmed by running qPCR amplification products on agarose gels; OCT4 ((A), 64bp), SOX2 ((B), 121bp), NANOG ((C), 91bp), KLF4 ((D), 110bp) and c-MYC ((E), 107bp). The reference genes GAPDH ((F), 122bp) and PUM1 ((G), 89bp) from cell lines were also checked. Ladder refers to the DNA marker in base pairs (bp); Lanes 1–5 refer to the cell line samples; Pos, positive control (NTERA-2 cell lines); NTC, no template control (RNase-free water) to confirm no contamination; No RT, reverse transcriptase negative control for primers that may detect genomic DNA. Figure S6. Full-length western blot images shown in Figure 7, of total protein extracted from four lung adenocarcino-ma-derived primary cell lines demonstrating protein expression of stemness-associated markers. Arrows indicate the presence of the proteins with expected band sizes for OCT4 (A), KLF4 (B) and c-MYC (C). NANOG (D) and SOX2 (E) were below detectable levels. Bands for α-tubulin (F) confirmed approximately equal protein loading.

## Patents

S.T., P.D. and T.I. are inventors of the patents of the patents Cancer Diagnosis and Therapy (PCT/NZ2015/050108; AUS/2012302419; JAP/2017528398; US/0281472), Cancer Therapeutic (PCT/NZ2018/050006), Novel Pharmaceutical Compositions for Cancer Therapy (PCT/NZ2019/050087).
