# Supplementary material for: Cell Populations Expressing Stemness-Associated Markers in Lung Adenocarcinoma
Source: Life (Basel). 2021 Oct 18;11(10):1106. doi: 10.3390/life11101106 (PMC8541371; doi:10.3390/life11101106)
Supplement: Supplementary file 1 [file life-11-01106-s001.zip › life-1370189-supplementary.pdf]

life

MDPI

Article

Cell Populations Expressing Stemness-Associated Markers in Lung Adenocarcinoma

Claudia Paterson [1], Ethan J Kilmister [1], Helen D Brasch [1], Nicholas Bockett [1], Josie Patel [1], Erin Paterson [1], Gordon Purdie [2], Sean Galvin [1,3], Paul F Davis [1], Tinte Itinteang [1] and Swee T Tan [1,4,5,*]

1 Gillies McIndoe Research Institute, Wellington 6242, New Zealand; claudiapatersonnz@gmail.com (C.P.); ethankilmister467@gmail.com (E.J.K.); helen.brasch@wellingtonscl.co.nz (H.D.B.); nick.bockett@gmri.org.nz (N.B.); josie.patel@gmri.org.nz (J.P.); erin.paterson@gmri.org.nz (E.P.); sean.galvin@ccdhb.org.nz (S.G.); paul.davis@gmri.org.nz (P.F.D.); tinte01@yahoo.com (T.I.)
2 Biostatistical Group/Dean's Department, University of Otago, Wellington 6021, New Zealand; gordon.purdie@otago.ac.nz
3 Department of Cardiothoracic Surgery, Wellington Regional Hospital, Wellington 6021, New Zealand
4 Wellington Regional Plastic, Maxillofacial & Burns Unit, Hutt Hospital, Lower Hutt 5010, New Zealand
5 Department of Surgery, The Royal Melbourne Hospital, The University of Melbourne, Melbourne 3010, VIC, Australia
* Correspondence: swee.tan@gmri.org.nz; Tel.: +64-4-2820366.

Supplementary Materials

Table S1. Patient characteristics and smoking status.

Patient	Age (years)	Gender	Ethnicity	Smoking Status
1	49	Male	NZ European	Current smoker
2	67	Female	NZ European	Ex-smoker
3	75	Male	NZ European	Non-smoker
4	77	Female	Pacific Island	Ex-smoker
5	62	Female	NZ European	Non-smoker
6	77	Female	NZ European	Ex-smoker
7	58	Male	Māori	Ex-smoker
8	72	Male	Pacific Island	Non-smoker
9	66	Female	Māori	Current smoker
10	67	Male	NZ European	Non-smoker
11	50	Female	NZ European	Current smoker
12	69	Male	NZ European	Non-smoker

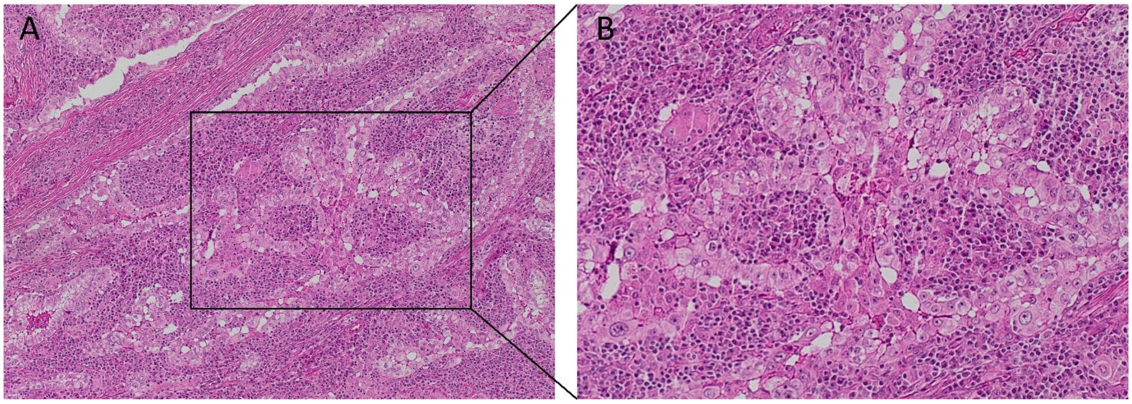

Figure S1. Representative hematoxylin and eosin images of lung adenocarcinoma demonstrating the tumor glands surrounded by stroma. Nuclei were counterstained with hematoxylin (**A**, **B**, blue). Original magnifications: 100× (**A**) and 400× (**B**).

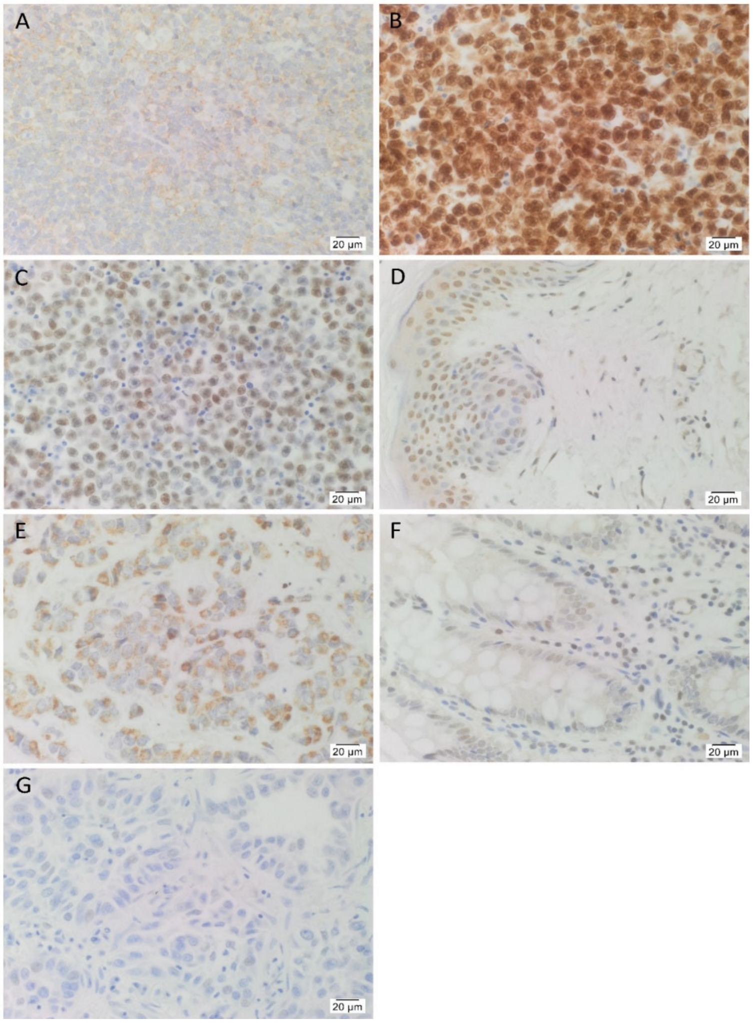

Figure S2. Representative positive control immunohistochemical-stained images demonstrating the expression of CD44 (**A**, brown) in tonsil, OCT4, (**B**, brown) and NANOG (**C**, brown) in seminoma, SOX2 (**D**, brown) in skin epidermis, KLF4 (**E**, brown) in breast carcinoma, and c-MYC (**F**, brown) in normal colon mucosa. A negative control (**G**). Nuclei were counterstained with hematoxylin (**A**-**G**, blue). Original magnification: 400×.

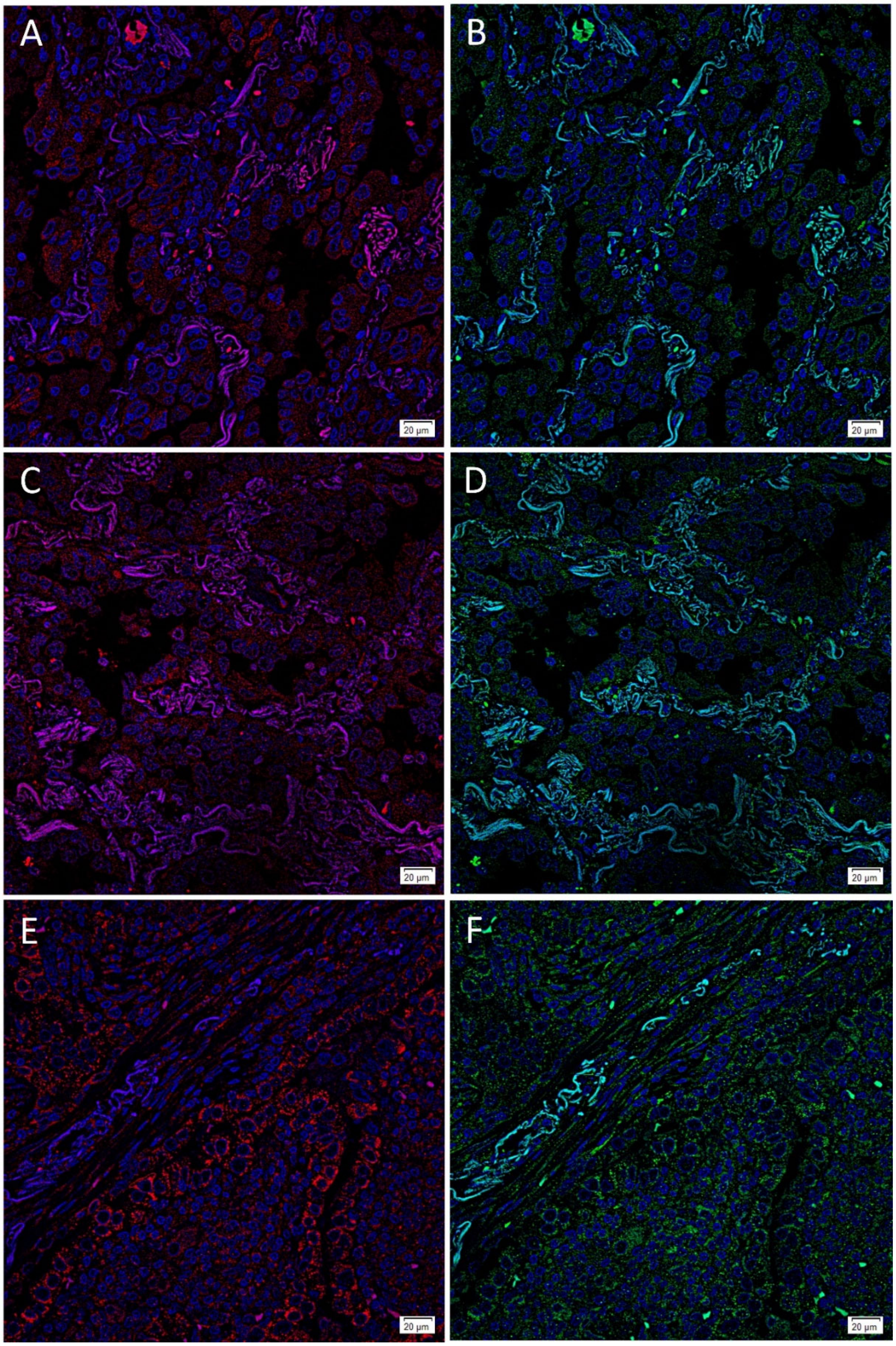
A
B
C
D
E
F
20 µm
20 µm
20 µm
20 µm
20 µm
20 µm

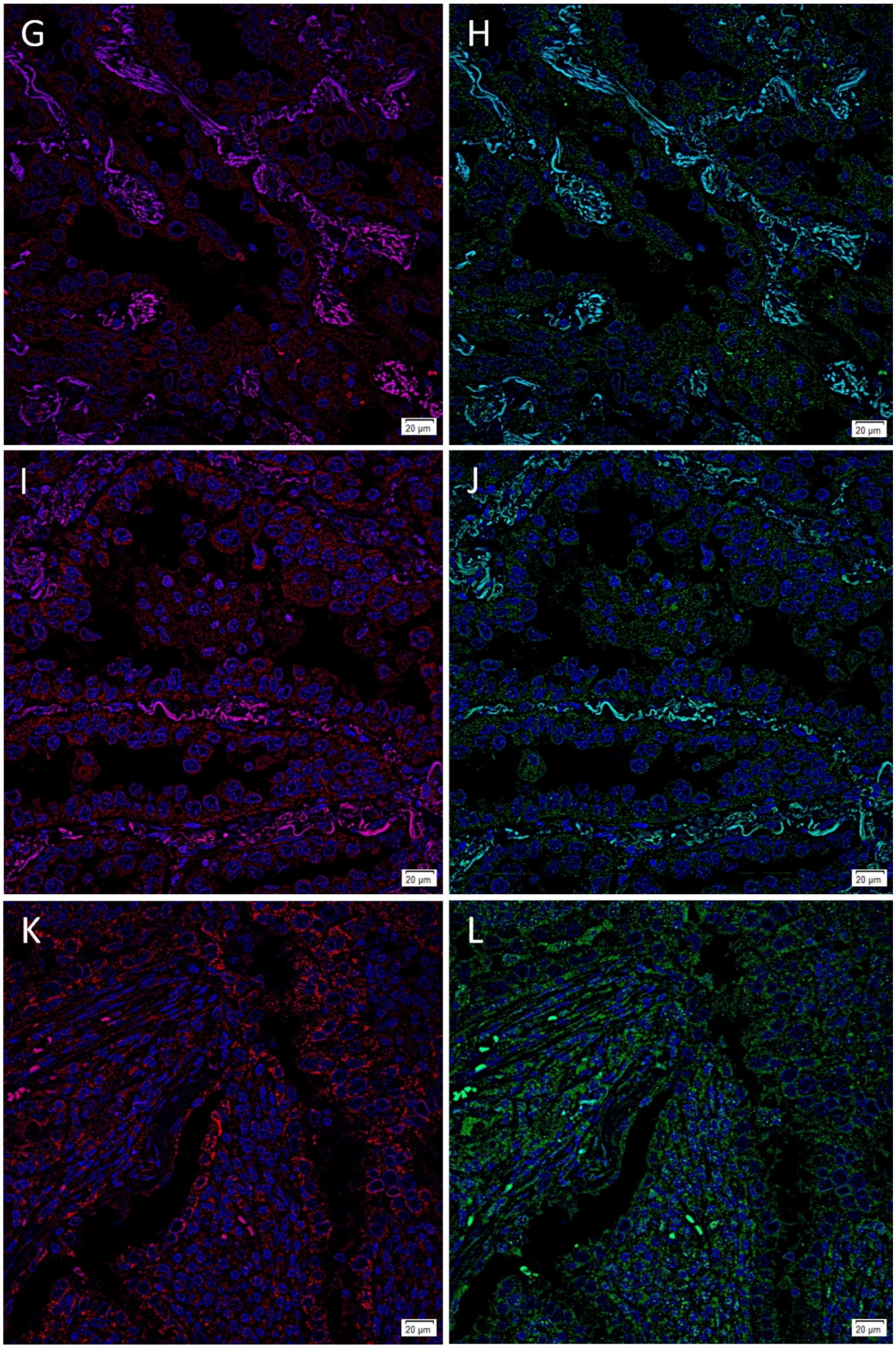
G
H
I
J
K
L
20 µm

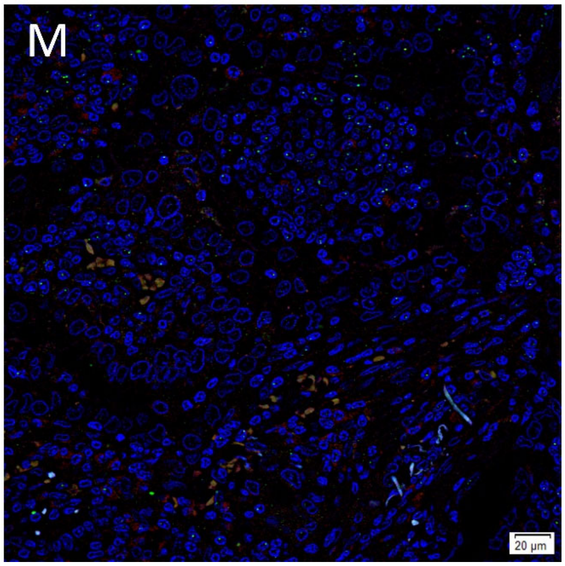

Figure S3. Split immunofluorescence-stained images shown in Figure 3 demonstrating the expression of NANOG (**A**, red) and OCT4 (**B**, green); SOX2 (**C**, red) and OCT4 (**D**, green); KLF4 (**E**, red) and OCT4 (**F**, green); NANOG (**G**, red) and c-MYC (**H**, green)**;** SOX2 (**I**, red) and c-MYC (**J**, green); KLF4 (**K**, red) and c-MYC (**L**, green). A negative control (**M**). Cell nuclei were counterstained with 4′,6 diamidino-2-phenylindole (**A**-**M**, blue). Original magnification: 400×.

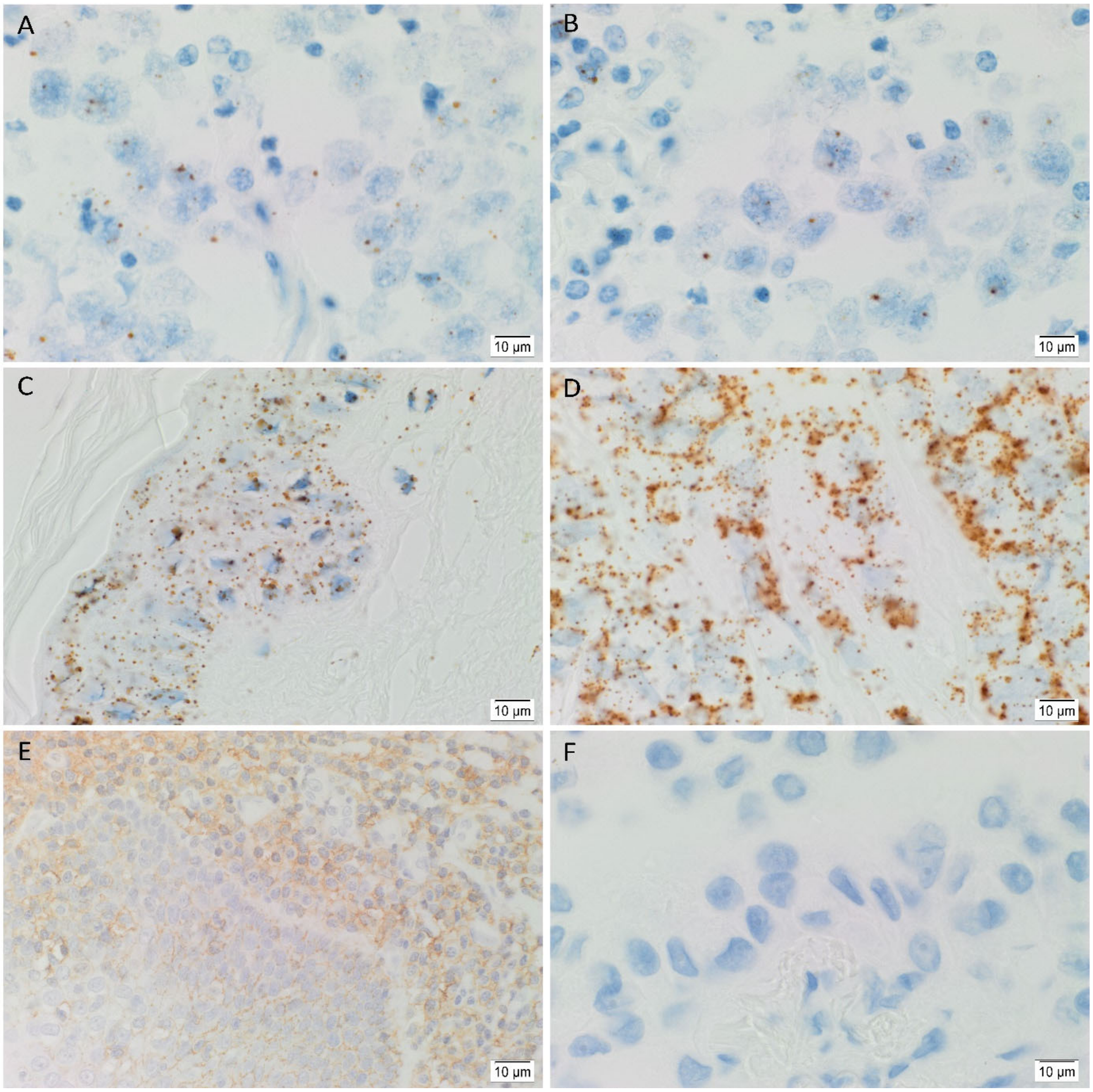

Figure S4. Representative *in situ* hybridization positive controls for OCT4 (**A**, brown) and NANOG (**B**, brown) in seminoma, SOX2 (**C**, brown) in normal skin, KLF4 (**D**, brown) breast carcinoma and c-MYC (**E**, brown) in normal prostatic tissue. Negative controls (**F**) performed on sections of lung adenocarcinoma tissue samples confirmed specificity of secondary antibody. Nuclei were counterstained with hematoxylin (**A**-**F**, blue). Original magnification: 1000×.

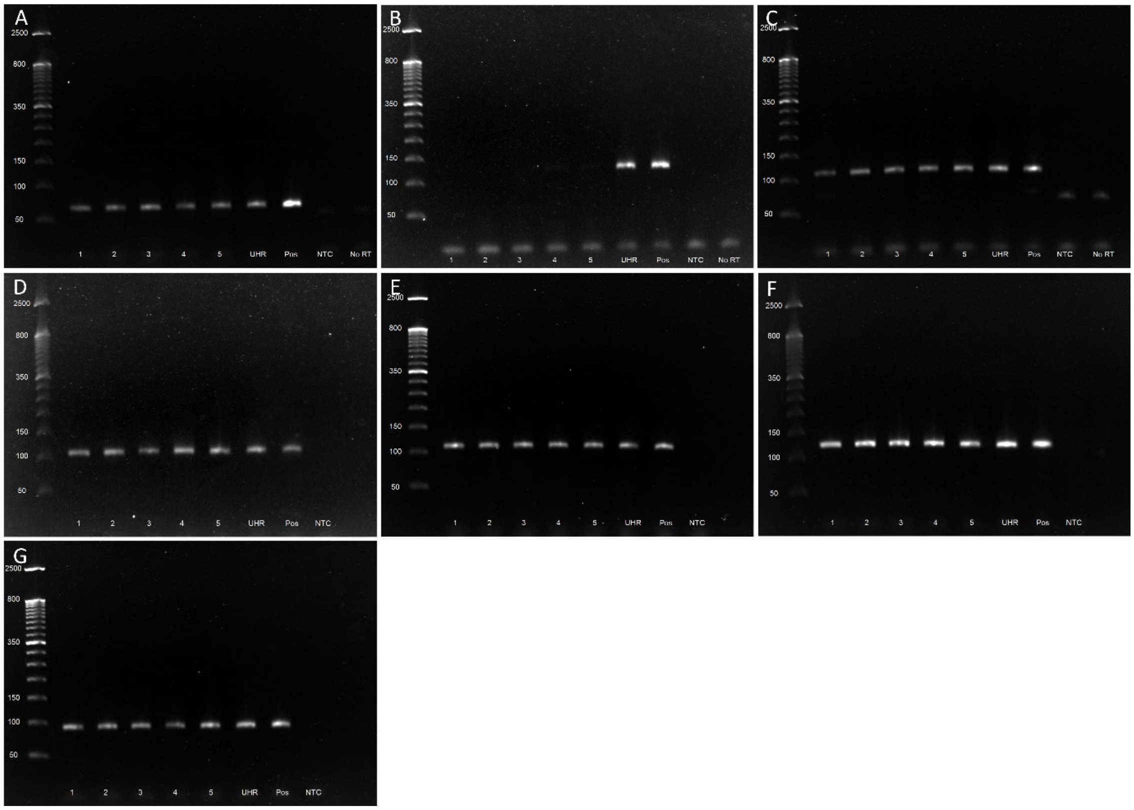

Figure S5. RT-qPCR amplification products from five lung adenocarcinoma-derived primary cell lines. The specificity of the primer probes was confirmed by running qPCR amplification products on agarose gels; OCT4 (**A**, 64bp), SOX2 (**B**, 121bp), NANOG (**C**, 91bp), KLF4 (**D**, 110bp) and c-MYC (**E**, 107bp). The reference genes GAPDH (**F**, 122bp) and PUM1 (**G**, 89bp) from cell lines were also checked. Ladder refers to the DNA marker in base pairs (bp); Lanes 1-5 refer to the cell line samples; Pos, positive control (NTERA-2 cell lines); NTC, no template control (RNase-free water) to confirm no contamination; No RT, reverse transcriptase negative control for primers that may detect genomic DNA.

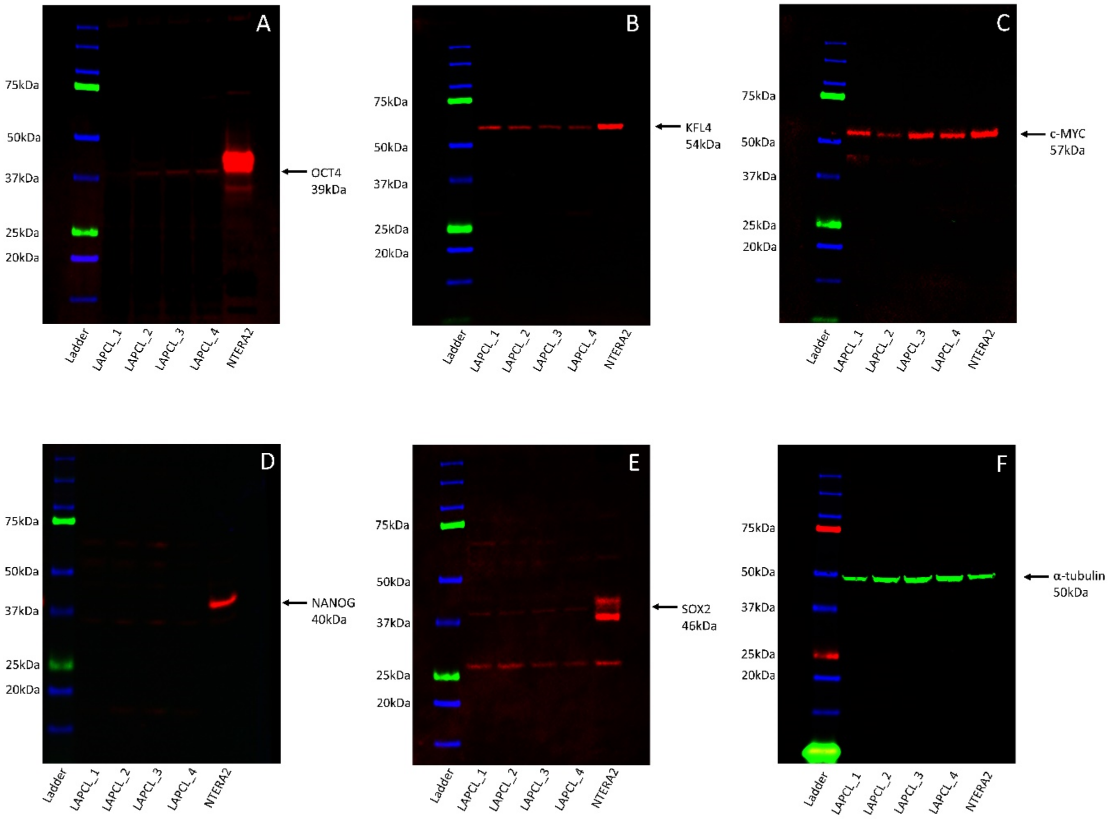

Figure S6. Full-length western blot images shown in Figure 7, of total protein extracted from four lung adenocarcinoma-derived primary cell lines demonstrating protein expression of stemness-associated markers. Arrows indicate the presence of the proteins with expected band sizes for OCT4 (**A**), KLF4 (**B**) and c-MYC (**C**). NANOG (**D**) and SOX2 (**E**) were below detectable levels. Bands for α-tubulin (**F**) confirmed approximately equal protein loading.
